# Headache prevalence in the population of L’Aquila (Italy) after the 2009 earthquake

**DOI:** 10.1007/s10194-011-0311-y

**Published:** 2011-02-18

**Authors:** Cristiana Guetti, Chiara Angeletti, Roberta Papola, Emiliano Petrucci, Maria Laura Ursini, Alessandra Ciccozzi, Franco Marinangeli, Antonella Paladini, Giustino Varrassi

**Affiliations:** 1 Department of Anaesthesiology, Intensive Care and Pain Medicine, University of L’Aquila, Viale San Salvatore, Edificio 6, 67100 L’Aquila, Italy; 2 VADO, Voluntary Association for Home Medical Care, L’Aquila, Italy

**Keywords:** Primary and secondary headache, Emergency setting, Pharmacological treatment

## Abstract

Stress induced by the events of daily life is considered a major factor in pathogenesis of primary tension-type headache. Little is known about the impact that could have a more stressful event, like a natural disaster, both in patients with chronic headache, both in people that do not had headache previously. The aim of the present study was to observe the prevalence of headache in the population following the devastating earthquake that affected the province of L’Aquila on April 6, 2009. The study population was conducted in four tent cities (Onna, Bazzano, Tempera-St. Biagio, Paganica). Sanitary access is recorded in the registers of medical triage, in the first 5 weeks, after the April 6, 2009. The prevalence of primary headache presentation was 5.53% (95% CI 4.2–7.1), secondary headache was 2.82% (95% CI 1.9–4.9). Pain intensity, assessed by Numerical Rating Scale score showed a mean value of 7 ± 1.1 (range 4–10). The drugs most used were the NSAIDs (46%) and paracetamol (36%), for impossibility of finding causal drugs. This study shows how more stressful events not only have an important role in determining acute exacerbation of chronic headache, but probably also play a pathogenic role in the emergence of primary headache. Also underlines the lack of diagnostic guidelines or operating protocols to early identify and treat headache in the emergency settings.

## Introduction

Headache accounts for about 1.2–4.5% of all accesses to emergency room (ER) in the adult population [1]. Secondary headache represents only 4.3–6.4% of cases [2]. Italian current data indicate that up to 23% of all neurological consultancies in ER may be related to clinical conditions characterized by headache [3]. Considering the high frequency of primary headache in ER during routine social sanitary activity, it is conceivable that the incidence of this condition may dramatically increase during catastrophic emergencies. After the earthquake of L’Aquila on April 6, 2009, Advanced Medical Presidiums (AMPs) were maintained in the region for a longer period than 72 h requested by law, because of the persistent difficulties in the sanitary organization. AMPs worked as ERs for patients affected by a large variety of pathological conditions, of different severity, including primary headache, either as symptom or disease in itself. As imaging techniques were not available for a diagnostic approach in the disaster area, medical history and accurate general and neurological examinations represented the most effective instruments for a correct diagnosis and exclusion of life-threatening conditions. Unfortunately, for the emergency physicians operating in- or extra-hospital setting, no guidelines or diagnostic algorithms are presently available for the diagnosis of primary headache including migraine, tension-type headache (TTH) and cluster headache, and more importantly for differentiating this condition from other organic causes of headache.

## Aims

The aim of the present observational study was to estimate the prevalence of primary and secondary headache in the population afferent to the four Advanced Medical Presidiums (AMPs), during the post-seismic emergency period. The secondary aim was to evaluate the frequency of use, types of pain killers and the short-term efficacy of the pharmacological treatment of the neurological pain.

## Materials and methods

The present observational study was carried out in four AMPs that were present in tent camps included in seven Mixed Operating Centers (MOC) operating in the area of L’Aquila during a 5-week period after the earthquake (from April 7 to May 11, 2009). AMP is a light pneumatic-tent structure, provided by the Department of Civil Defense, where a voluntary staff of doctors (2–3 MD/day) and nurses (2–3 Nurses/day) operates. The staff was on duty shifts of 8–12 h and provided healthcare assistance to the population. For triage, conventional emergency codes were employed:

- WHITE = No emergency
- GREEN = Secondary emergency
- YELLOW = Primary emergency
- RED = Extreme emergency
- BLACK = Death.

Demographic parameters, including name, surname, gender, age, physical conditions (based on a two points scale: 1 = self-sufficient, 2 = not self-sufficient) have been registered for each patient. Also cardio-respiratory parameters, including blood pressure (BP), heart rate (HR), body temperature [BT (in °C)] and oxygen saturation (SpO_2_) have been registered. State of consciousness by Glasgow Coma Scale (GCS) and pain intensity during cephalalgic crisis (time zero, *T0*) by the verbal numerical rating scale (NRS) have been assessed. Neurological examination has been performed at baseline. Associated clinical conditions and previous and/or current therapy have been registered, including allergies, tobacco addiction, alcoholism and drug addiction. Finally, diagnosis of primary or secondary headache was made on the basis of a simple questionnaire (Table 1) and a therapeutic treatment was defined. The early response to treatment was evaluated by the NRS score at *T*_2h_ = 2 h after drug administration, when the patient was still under medical control in the AMP setting; NRS score was reevaluated after 24 and 48 h (*T*_24h_ and *T*_48h_) after drug administration for a short-term follow-up. The AMP centers were located in Bazzano, Tempera-S.Biagio, Onna and Paganica. Anesthetists in a common registry have collected data. All patients, including civilians, Civil Defense volunteers, Security force, soldiers and firemen afferent for the first time to the health structures have been considered for the study. Data are presented as mean and standard deviation, frequencies and prevalences in percentage. Statistical analysis used repeated-measures for NRS score analysis of variance (RM-ANOVA) and Bonferroni *t* test (all pairwise multiple comparison) as least significant difference test. *P* value of <0.05 was considered statistically significant.

**Table 1:** Diagnosis of primary or secondary non traumatic headache (NT) by a simple questionnaire

Diagnostic questionnaire for headache NT
1. It is the first time you have headache? This is unusual headache, the most intense of which has ever suffered?
2. As the headache started?
3. Is there something that triggered the headache?
4. Where is localized the pain?
5. How intense is this headache (NRS score)?
6. What other symptoms is associated with headache?
7. How long have you suffer from headaches?

## Results

**Fig. 1:**
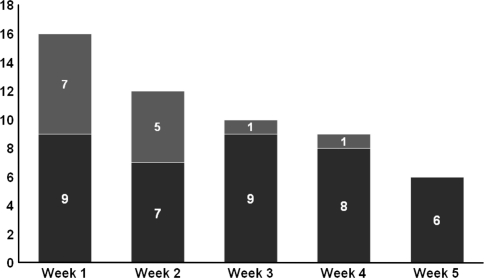
Weekly accesses to AMPs. *Black column* represented patients already suffering from primary headache, *grey column* represented subjects hitherto not-headache

**Fig. 2:**
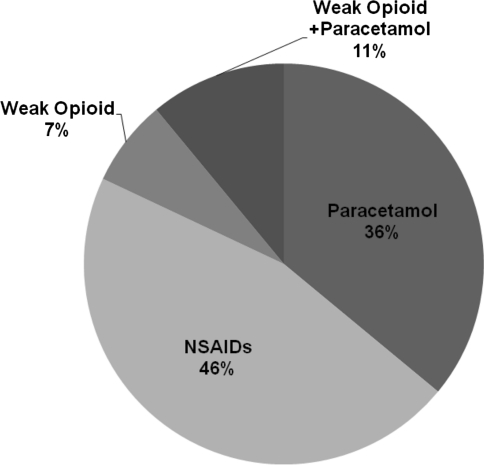
Drugs administered for the treatment of headache (frequency of use, %)

**Fig. 3:**
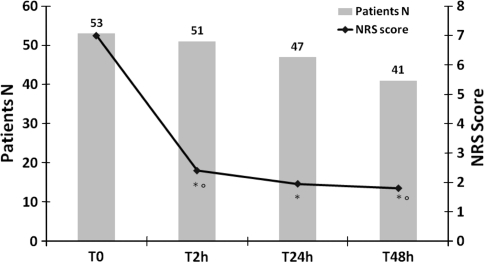
Trend of NRS score in the first 48 h after treatment. In *columns* are represented the number of patient. Data are presented as mean ± SD and percentage; *P < 0.001 versus *T0*; °*P* = 0.006 *T*_2h_ versus *T*_48h_

The population living in the four tent camps included 1,777 civilians and 635 volunteers, for a total population of 2,412 persons. A total of 53 cases of primary headache have been registered among the first accesses to the AMPs triage managed by the personnel of the civil defense and voluntary associations and by physicians from the University of L’Aquila, department of Anesthesiology, Intensive Care and Pain Therapy during the 5-week period from April 7 to May 11, 2009, immediately after the April 6, 2009 earthquake of L’Aquila. The prevalence of primary headache was 5.53% (95% CI 4.2–7.1), among the 958 first accesses to AMP, whereas secondary headache was 2.82% (95% CI 1.9–4.9) (Table 2). Mean age of the patients was 43.2 ± 16 (range 19–89 years), females were 31 (58.5%) and males 22 (41.5%). Primary headache was 16.6% of all painful pathological conditions treated. Figure 1 shows the time course of primary headache during the 5-week observation period: a higher prevalence is evident during the first 3 weeks. Episodes of relapsed headache represented 26% of cases (*n* = 14), while the first episode was present in 74% of patients (*n* = 39). A quantitative estimate of pain intensity by the NRS scale at *T0* showed an average intensity of 7 ± 1.1 (range 4–10). Antinflammatory drugs have been administered in 46% of cases, paracetamol in 36%, the association of weak opioids plus paracetamol in 11%, weak opioids in 7% (Fig. 2). The intensity of pain significantly decreased at time *T*_2h_, *T*_24h_ and *T*_48h_; NRS score, indeed, was 2.4 ± 0.9 after 2 h (*T*_2h_), this means a 5-point difference in the NRS scale (*P* < 0.001; 95% CI) and a 75% decrease compared to *T0*. Pain was controlled by pharmacological treatment in the following 2 days of observation. The percentage of patients on symptomatic treatment was 89% at *T*_24h_ with a NRS score of 1.95 ± 0.75 and 77% at *T*_48h_. The average NRS score at *T*_48h_ was 1.8 ± 0.68 (Fig. 3).

**Table 2:** The prevalence of primary and secondary headache registered among the first accesses to the AMPs triage in the first 5 weeks after earthquake

Primary headache	*N*	Secondary headache	*N*
1. Migraine	6	5. Headache attributed to head and/or neck trauma	5
1.1 Migraine without aura		5.1 Acute post-traumatic headache	
1.2 Migraine with aura		5.6 Headache attributed to other head/neck trauma	
1.6 Probable migraine			
2. Tension-type headache (TTH)	37	6. Headache attributed to cranial or vascular disorder	3
2.1 Infrequent episodic tension-type headache			
2.2 Frequent episodic tension-type headache			
2.3 Chronic tension-type headache			
2.4 Probable tension-type headache			
3. Cluster headache and other trigeminal autonomic cephalalgias	2	7. Headache attributed to non vascular intracranial disorder	1
		7.6 Headache attributed to epileptic seizure	
4. Other primary headache	8	8. Headache attributed to a substance or its withdrawal	1
4.1 Primary stabbing headache			
4.2 Primary cough headache			
		10. Headache attributed to disorder of homoeostasis	11
		10.3 Headache attributed to arterial hypertension	
		10.4 Headache attributed to hypothyroidism	
		10.7 Headache attributed to other disorder of homoeostasis	
		11. Headache or facial pain attributed to disorder of cranium, neck,	3
		Eyes, ears, nose, sinuses, teeth, mouth or other facial or cranial	
		Structures	
		12. Headache attributed to psychiatric disorder	2
Total	53		27
Prevalence (%)	5.53		2.82

Source: The International Classification of Headache Disorders, 2nd Edition (ICHD-II) 2004

## Discussion

The large majority of primary headache in patients afferent to ER are of essential origin. In fact, up to 90% of patients suffering from headache are affected by the TTH or by migraine [4]. The peculiar clinical context of the present observational study may provide some interesting clues. The population study was stratified by gender and age according to the epidemiological studies on primary headache reported in the literature [5]. The different types of primary headache were distributed according to the prevalence observed in the general population [6]. However, a substantial increment up to 70% of TTH forms was observed when compared with the 11% migraine and 20% remaining types (cluster, trigeminal, etc.). Secondary forms represented only 2.82% of the total, and were correctly diagnosed on the basis of the reported questionnaire and the accurate observation of the associated symptoms; obviously, these cases required a different therapeutic approach when compared with primary headache. The prevalence of primary headache was high, reaching a 16% of all post-seismic painful pathologies; also in relation to pathologies of other origin, primary headache represented the 5.53% of all causes of access to AMPs within the 5 weeks of the study. The shortage of diagnostic tools, including routine chemistry and imaging techniques, did not prevent a correct diagnosis of primary conditions that was mostly based on the exhaustive differential diagnosis. During a natural disasters, the clinical presentation of headache is super imposable in most of the cases; symptoms may be associated or masked by multiple external factors, including fasting, dehydratation, insomnia or panic. Overall, headache episodes may be induced by the stress related to the catastrophic event. A stressful event, indeed, has been shown to precipitate a pain episode of TTH or migraine [7]. It has been hypothesized that a chronicizing stress, poor stress tolerance, prolonged physiological response to stressors or insufficient recovery from stress can cause headache, chronic pain and multiple physical disturbances [8]. These factors support the observations of the present study. Several stress-related factors may have induced or worsened the episodes of headache. First of all, the uncomfortable life conditions, including living in tents, atmospheric agents, high temperature excursion (hot days, wintry nights and/or rain), small uncomfortable beds, hard physical work in order to meet personal and community daily life needs in the emergency centers. A drastic interruption of domestic and social habits as a consequence of the catastrophic event caused a deep sense of impotence and limitation of autonomy that seriously influenced individual and community mood [9]. The time course and distribution of cases during the 5-week observation period shows that inadequate adaptation to multiple acute stressors directly or indirectly related to disaster played a key role in inducing headache episodes. The increased prevalence of primary headache, indeed, during 3 weeks after the earthquake may be related to the stress of the acute event and the associated factors including psycho-physical changes of individuals, due to acceptance, hope, resignation or other factors such as progressive improvement of social, hygienic and structural conditions and decreased intensity of the seismic swarm.

The high frequency of first episodes of primary headache is another distinctive element that underlines the importance of chronicizing stress in the pathogenesis of this condition. The “central” mechanisms of the disease may have been triggered off, in particular, by peripheral mechanisms such as contraction, hypersensitivity, pain of pericranial and cervical muscles, secondary to the above-mentioned hard life context. Overall, these elements may be responsible for increase of chronic forms after the catastrophic event [10]. Several studies, indeed, have shown that activation of muscles of the pericranial areas related to pain may be induced directly by stress or by modulation of specific nociceptive afferences related to episodes of central sensitisation. In fact, central sensitisation is recognized as an important mechanism in the pathogenesis of primary headache, either TTH or migraine [11–13]. In the presence of a natural disaster, the relationship between stress and pain may perpetuate a dangerous vicious circle. The physiopathological mechanisms of headache may have amplified the role of the stressful event in cases exposed and stress, in turn, may have enhanced the relapse and/or appearance of pain. This vicious circle should be blocked also in emergency situations by the administration of effective analgesic drugs, in order to prevent pain chronicization, in particular, in post-traumatic cases. In the present study, drugs most frequently administered as pain relievers included paracetamol (36%) and non-steroid antiinflammatory drugs (46%); weak opioids (18%), either alone or associated with paracetamol were used in a smaller percentage of cases. The high intensity of pain (average NRS score 7 ± 1.1, severe pain) in the acute phase of headache often required a strict monitoring of vital parameters [BP, HR, BT (in °C), SpO_2_] and of the analgesic effect of drugs by the NRS score during the following 2 h. The decrease of pain intensity as assessed by the difference between NRS scores was the reference parameter for estimating the efficacy of drugs either immediately (*T*_2h_ = first 2 h) as in the following 24–48 h (*T*_24h_ and *T*_48h_). It is known that about two-third of patients complain new episodes of pain within 24 h after discharge from ER; in half of them, the intensity of pain is mild-severe [14]. Up to 50% of patients report a functional disability within 24 h after the headache crisis causing the access to the ER [15]. In the present study, 77% of patients required the administration of analgesic drugs up to 48 h after the onset of the crisis in order to control pain. This suggests that the mechanisms triggering and maintaining headache were operating for a longer period than the stress-induced peripheral and muscular mechanisms usually do. An early treatment, although with the limited number of drugs available, and a strict monitoring of patients, allowed us to substantially control pain, as shown by the decrease of the average NRS scores within 48 h (*T*_48h_ **=** 1.8 ± 0.68). Figures 1, 2, 3 shows the time course of pain during the observation period. The choice of the drug, in the large majority of cases NSAIDs or paracetamol, according to medical history and characteristic of pain, was mostly influenced by the shortage of specific drugs, such as triptans [16], ergot derivatives [17], antiepileptic drugs [18], and narcotic analgesics [19]. Analgesic drugs have been mainly administered orally; the oral route facilitated the therapeutic management of patients after discharge and improved their compliance to treatment during the following 48 h.

## Conclusions

The present observational study has been markedly influenced by the adverse clinical setting in which it has been carried out and by the multifactorial pathogenesis of headache. The most important aspect of the study is that the observation of patients was protracted for 48 h, in a clinical condition characterized by shortage of sophisticated diagnostic instruments. The first steps for identifying primary headache in patients afferent to AMPs included an accurate medical history, a short questionnaire (no approved questionnaires for headache in ERs are available) and physical examination. Valuable information has been derived from vital parameters, such as body temperature, arterial blood pressure, cardiac frequency and NRS score. Further important diagnostic elements have been derived from physical examination, including palpation of the aching head and neck areas, and complete neurological examination. These simple elements allowed us to formulate a correct diagnosis and organize a therapeutic intervention for the following 48 h; this approach obtained a substantial control of pain in all primary forms. Specific potent drugs, including triptans and narcotic analgesics, were unavailable in our setting; this reveals a poor sanitary education and care in the treatment of headache and, more generally, of pain syndromes in emergency situations. This last consideration is of major concern due to the relevant prevalence of headache in natural disaster setting, the prognostic severity of secondary forms and the high risk of chronic headache.
